# Treatments of unruptured brain arteriovenous malformations: A systematic review and meta-analysis

**DOI:** 10.1097/MD.0000000000026352

**Published:** 2021-06-25

**Authors:** Renjie Liu, Yongle Zhan, Jianmin Piao, Zhongxi Yang, Yun Wei, Pengcheng Liu, Xuan Chen, Yu Jiang

**Affiliations:** a Department of Neurovascular Surgery, The First Bethune Hospital of Jilin University, Changchun 130021, Jilin Province; b Department of Epidemiology and Biostatistics, School of Population Medicine and Public Health, Chinese Academy of Medical Sciences and Peking Union Medical College, Beijing 100730, China.

**Keywords:** complications, meta-analysis, obliteration, treatment, unruptured brain arteriovenous malformations

## Abstract

**Background:**

The best therapeutic option for unruptured brain arteriovenous malformations (bAVMs) patients is disputed.

**Objective:**

To assess the occurrence of obliteration and complications of patients with unruptured bAVMs after various treatments.

**Methods:**

A systematic literature search was performed in PubMed, EMBASE, Web of Science, and so on to identify studies fulfilling predefined inclusion criteria. Baseline, treatment, and outcomes data were extracted for statistical analysis.

**Results:**

We identified 28 eligible studies totaling 5852 patients. The obliteration rates were 98% in microsurgery group (95% confidence interval (CI): 96%–99%, *I*^2^ = 74.5%), 97% in surgery group (95%CI: 95%–99%, *I*^2^ = 18.3%), 87% in endovascular treatment group (95%CI: 80%–93%, *I*^2^ = 0.0%), and 68% in radiosurgery group (95%CI: 66%–69%, *I*^2^ = 92.0%). The stroke or death rates were 1% in microsurgery group (95%CI: 0%–2%, *I*^2^ = 0.0%), 0% in surgery group (95%CI: 0%–1%, *I*^2^ = 0.0%), 4% in endovascular treatment group (95%CI: 0%–8%, *I*^2^ = 85.8%), and 3% in radiosurgery group (95%CI: 3%–4%, *I*^2^ = 82.9%). In addition, the proportions of hemorrhage were 2% in microsurgery group (95%CI: 1%–4%, *I*^2^ = 0.0%), 23% in endovascular treatment group (95%CI: 7%–39%), and 12% in radiosurgery group (95%CI: 12%–13%, *I*^2^ = 99.2%). As to neurological deficit, the occurrence was 9% in microsurgery group (95%CI: 6%–11%, *I*^2^ = 94.1%), 20% in surgery group (95%CI: 13%–27%, *I*^2^ = 0.0%), 14% in endovascular treatment group (95%CI: 10%–18%, *I*^2^ = 64.0%), and 8% in radiosurgery group (95%CI: 7%–9%, *I*^2^ = 66.6%).

**Conclusions:**

We found that microsurgery might provide lasting clinical benefits in some unruptured bAVMs patients for its high obliteration rates and low hemorrhage. These findings are helpful to provide a reference basis for neurosurgeons to choose the treatment of patients with unruptured bAVMs.

## 1 Introduction

Unruptured brain arteriovenous malformations (bAVMs) is a complex vascular disease with estimated annual hemorrhage rates of 1%–4%. It presents a substantial burden for patients because of the potential for serious neurological complications or death.^[1,2]^ Even though the annual hemorrhage rate is relatively low, the mortality rate after the first hemorrhage is as high as 10%. After 3 months, there is an additional 20% mortality with patients who survive.^[3,4]^ The increasing use of imaging technology has increased the detection rates of unruptured bAVMs and has inevitably raised the question of their treatments.

The treatments of unruptured bAVMs include surgical resection, radiosurgery, endovascular treatment, medical management group, or any combination of the modalities above. However, different treatments may cause related complications in various degrees. Mohr et al^[5]^ conducted a randomized trial of unruptured cerebral arteriovenous malformation (ARUBA) and found that in terms of death or stroke prevention, the medical management group was superior to the treatment group. However, the potential long-term benefits from intervention could not be estimated on account of the early termination of the trial.^[6]^ Meanwhile, Rutledge et al^[7]^ found that the risk of stroke or death (HR: 1.34, 95%CI: 0.12–14.53, *P* = .81) and degree of clinical impairment among patients with treatment group were lower than that of the previous study. Endovascular treatment, less invasive treatment for vascular diseases, is now widely applied worldwide. However, as a stand-alone treatment, it has been shown to be generally incomplete. A trial showed 117 bAVMs patients were treated with Onyx embolization, in which, only 23 patients achieved complete obliteration.^[8]^ Radiosurgery, although effective in many cases with less common short-term risks than other treatment modalities, requires a few years to achieve nidus obliteration, and the risk of hemorrhage can be slightly increased during the period between treatment and obliteration.^[9]^ Moreover, the longer, continuous risks of radiation, which may occur even >10 years after treatment, are still largely unknown.^[2]^ Microsurgery, as an essential treatment of unruptured bAVMs, provides immediate benefits and eliminates the risk of further lifetime hemorrhage.^[10]^ A study showed that good outcomes could be achieved by microsurgical resection in patients with Spetzler–Martin grade I–II unruptured bAVMs.^[11]^ However, it is challenging to treat patients with S–M grade IV–V unruptured bAVMs. Therefore, the best therapeutic option for unruptured bAVMs patients is still a controversial subject among cerebrovascular neurosurgeons.^[11]^

At present, there is lacking of high-quality meta-analysis concerning which treatment is better for unruptured bAVMs. And the debate exists in the most favorable therapeutic approach. Furthermore, most patients diagnosed with unruptured bAVMs experience partially disabling symptoms including seizures and headaches, which can reduce their quality of life to some extent.^[12]^ Therefore, we conducted a meta-analysis to explore the best treatment for unruptured bAVMs patients and to evaluate these methods.

In this systematic review, we systematically collected all relevant evidence and conducted a meta-analysis to reported the combined obliteration rates of obliteration, stroke or death, hemorrhage, and neurological deficit after microsurgery, endovascular treatment, surgery, or radiosurgery. The present study aims to investigate the advantages and disadvantages among different treatments for unruptured bAVMs patients, and to provide a more reliable basis for neurosurgeons to choose clinical treatments.

## 2 Methods

### 2.1 Literature search and inclusion criteria

We conducted this systematic review and meta-analysis following the Preferred Reporting Items for Systematic Reviews and Meta-Analyses Statement. Ethical approval is inapplicable for this study. A systematic literature search was performed through April 1, 2020, in PubMed, EMBASE, Web of Science, and clinical trials registration system (http://www.ClinicalTrials.gov) and so on with the following MeSH terms: unruptured bAVMs (unruptured intracranial arteriovenous malformations/unruptured brain arteriovenous malformation) AND terms for treatment (surgery or microsurgery or radiosurgery or endovascular treatment or embolization/artificial embolism).

After screening by title and abstract, only studies that met the following criteria could be included: (1) original articles with full text; (2) patients with unruptured bAVMs received 1 kind of treatments; and (3) reported the proportion of at least 1 outcome, such as obliteration, stroke/death, hemorrhage, neurological deficit, and so on. We have excluded studies with overlapping published data from the same institution. Review articles, letters, editorials, comments, case reports, and technical reports were excluded. What's more, studies reporting other kinds of vascular malformations including ruptured bAVMs, dural arteriovenous fistula, and cavernous malformations were also excluded. All retrieved articles were independently screened by 2 authors (RJL and YLZ) according to the above selection criteria, and discrepancies on whether to include a study were resolved by discussion.

### 2.2 Data extraction

One author (RJL) extracted the following data, when available, from selected studies of the included articles: a total number of patients, average age, mean or median duration of follow-up, proportions of obliteration, annual hemorrhage, neurological deficit, stroke, or death, functional outcomes, radiation-induced change (RIC) (radiologic RIC, symptomatic RIC, and permanent RIC), and other complications after treatments. To ensure accuracy, another 2 authors (YLZ and JMP) reviewed the data collected and formatted separately and compared it with the source material. Disagreements on data extraction were resolved through consensus and, if needed, by consulting the senior author (XC and YJ).

### 2.3 Quality assessment

Two authors (ZXY and YW) independently assessed the risk of bias and graded the quality of all the included studies. The Cochrane risk of bias tool,^[13]^ Methodological Index for Non-Randomized Studies,^[14]^ and Newcastle–Ottawa Scale^[15]^ were used to assess the bias and quality of randomized controlled trials (RCTs), non-RCTs, and cohort studies, respectively. Studies with higher scores represented higher quality.

### 2.4 Statistical analysis

The pooled rate with its 95% confidence interval (CI) was the effect size to estimate the proportion of obliteration and complications after treatment. Inter-study heterogeneity was assessed by statistics with significance set at the *P* < .10 level. We also applied the *I*^2^ statistic to estimate the heterogeneity as low (<25%), moderate (25%–75%), and high (>75%). Effect size with its 95%CI was calculated with a random-effects model when heterogeneity was considerable among studies; otherwise, the fixed-effects model was used. Subgroup analyses were conducted stratified by continent, study duration, time of follow-up, study quality, study design, and publication characteristics. To evaluate the impact of different subgroups on the pooled effect estimates, meta-regression was additionally performed. What's more, sensitivity analyses were also conducted to evaluate whether any single study dominated the results of the meta-analysis when the pooled results had high heterogeneity. Finally, we constructed funnel plots and Egger tests to assess the possibility of publication bias for the main outcomes with significance at the *P* < .05. All the statistical analyses were performed using STATA 15.0 (Stata Corporation, College Station, TX, USA).

## 3 Results

### 3.1 Description of the included studies

**Figure 1:**
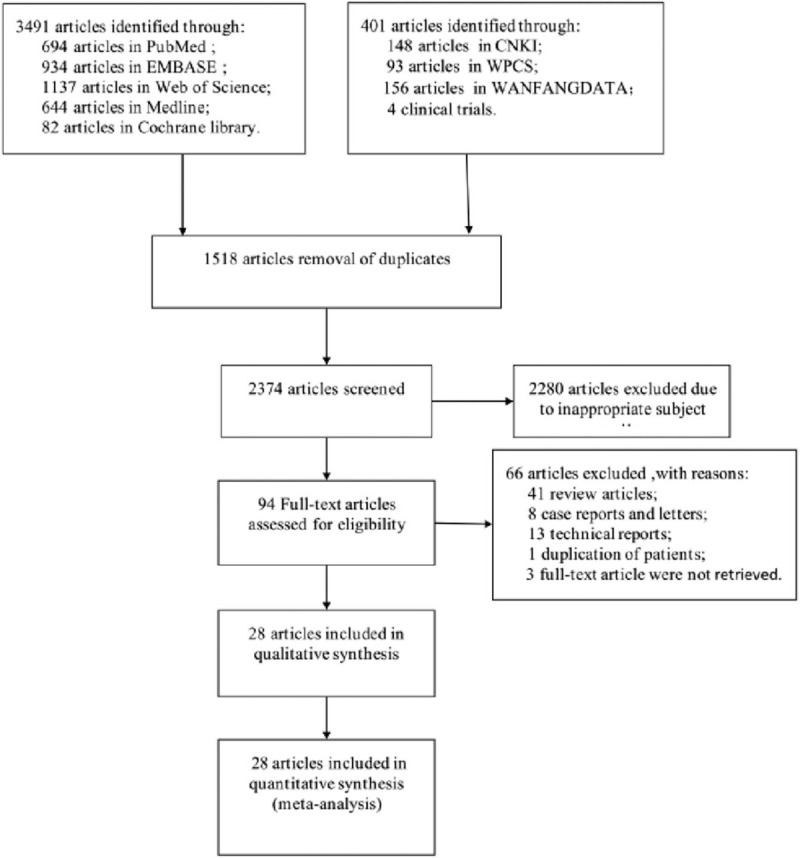
Flowchart demonstrating the literature review process. After duplication-checking, title and abstract screening, and full-text review, 28 studies fulfilled the predefined inclusion criteria and were included in the meta-analysis.

The steps of our systematical search were shown in Figure 1. Through a systematical literature search, a total of 3892 potentially relevant studies were identified. After duplication-checking, title and abstract screening, and full-text review, 28 full-text studies^[1–3,5,7,11,16–37]^ including 5852 patients were included in the final analysis. The characteristics of the included studies were summarized in Table 1. The present meta-analysis included 4 RCTs, 16 non-RCTs, and 8 cohorts studies, and 11 studies reached high quality. All studies were published between 2012 and 2020, among which, 18 were conducted in North America, 6 in Asia, and 4 in Europe. Duration and follow-up of the included studies ranged from 1989 to 2017, and 6 days to 14.1 years. As to types of treatment, 16 studies reported on radiosurgery, 4 on microsurgery, 9 on endovascular treatment, and 4 on surgery. All the included studies looked for outcomes such as obliteration rate and complications after treatment.

**Table 1:** Baseline characteristics.

									Spetzler–Martin grade (n)						
Study	Location of study	Year	Study design	Follow-up	Age (y)	Gender	Simple size (n)	Therapy	I	II	III	IV	V	Outcomes (n)	Quality evaluation
Qureshi 2020^[14]^	US		RCT	11.8 ± 9.4 mo	43.7 ± 11.1 yr	Male (n = 14)	26	Endovascular treatment	7	10	8	1		Stroke, death	High
Chen 2020^[13]^	US	1987–2014	Cohort study	80.8 ± 59.4 mo	13.5 ± 3.7	Male (n = 62)	101	Radiosurgery	9	39	36	14	3	Hemorrhage, death, permanent RIC, obliteration, radiological RIC, symptomatic RIC	High
Ozpar 2019^[15]^	Turkey	2008–2016	NRCT				26	Endovascular treatment						Intracranial hemorrhage, neurologic deficits, death	Medium
Chen 2019^[16]^	US	20012013	Cohort study	8.7 ± 13.2 mo	35.9 ± 14.1	Male (n = 6)	15	Microsurgery	2	8	3	2	0	Obliteration, neurologic deficits, intracranial hemorrhage	High
				97.0 ± 54.8 mo	38.7 ± 14.1	Male (n = 9)	19	Radiosurgery	2	11	4	2	0		
Baharvahdat 2019^[17]^	France	2005–2015	NRCT	11.3 ± 13.8 mo	38.5 ± 9.9	Male (n = 46)	88	Endovascular treatment	31	57				Obliteration, death, mRS score	High
Karlsson 2018	Singapore		Cohort study	6.5 (0.1–25) yr	42 (8–80)	Male (n = 763)	1351	Radiosurgery						Obliteration, hemorrhages, death, neurological deficit	Medium
Link 2018^[3]^	US	2004–2017	NRCT	33.2 mo	43 ± 1.3	Male (n = 45)	2	Surgery	16	35	29	6	0	Obliteration, stroke or death, neurologic deficits, long-term clinical impairment	Medium
							9	Radiosurgery							
							8	Endovascular treatment							
Nerva 2018^[20]^	US	2005–2012	NRCT	3.5 ± 1.9 yr	39.9 ± 19.6	Male (n = 29)	41	Radiosurgery	4	13	11	10	3	Obliteration. intracranial hemorrhage, neurologic deficits	Medium
Yang 2018^[19]^	US	2009–2013	NRCT	7.7 ± 10.8 yr	40.2 ± 19.5		32	Endovascular treatment						Intracranial hemorrhage	High
Wong 2017^[1]^	Canada	1994–2014	NRCT	36.1 mo	38.5	Male (n = 67)	155	Microsurgery	52	66	30	7	0	Obliteration, intracranial hemorrhage, neurologic deficits	Medium
Tong 2017^[5]^	China	2008–2015	NRCT	50.0 ± 23.2 mo	30.5 ± 12.2	Male (n = 187)	282	Microsurgery	186	72	24	obliteration, seizure outcome, neurological deficits, intracranial hemorrhage, hydrocephalus, infections, mRS score	Medium		
Starke 2017^[21]^	US		Cohort study	81.1 ± 55.5 mo	12.6 ± 3.7		112	Radiosurgery						Obliteration, intracranial hemorrhage, radiologic RIC, symptomatic RIC, permanent RIC	Medium
Ding 2017^[24]^	US		NRCT	90.5 ± 63.3 mo	41.8 ± 14.2	Male (n = 187)	232	Radiosurgery	49	183				Obliteration, stroke or death, intracranial hemorrhage, Radiologic RIC	Medium
Ding 2017^[25]^	US		Cohort study	33.0 mo	34.7 (3.5–81.1)	Male (n = 483)	938	Radiosurgery	84	323	436	89	6	Obliteration, intracranial hemorrhage, symptomatic RIC, Permanent RIC, death	Medium
Singfer 2017^[22]^	US	2001–2014	NRCT	60.0 mo	38.0 ± 11.0	Male (n = 32)	61	Endovascular treatment	11	20	21	8	1	mRS score, stroke or death	Medium
Mohr 2017^[23]^	US	2007–2013	RCT	33.3 (16.3–49.8) mo	>18		114	Endovascular treatment						Functional impairment	High
Javadpour 2016^[26]^	UK	2004–2014	Cohort study	69 (6–126) mo		Male (n = 16)	34	Microsurgery	8	16	8	2		Death or stroke, neurological deficit, mRS score	High
Hanakita 2016^[2]^	Japan	1990–2010	NRCT	62 (36–102) mo	39 (30–50)	Male (n = 151)	240	Radiosurgery	76	95	53	11	5	Obliteration, intracranial hemorrhage, neurological deficits, seizures, hemiparesis, dysesthesia, hemianopia, aphasia	Medium
Ding 2016^[27]^	US	1997–2014	NRCT	86.2 ± 62.3 mo	39.7 ± 13.7	Male (n = 271)	509	Radiosurgery	49	183	245	32		Obliteration, intracranial hemorrhage, symptomatic RIC, permanent RIC, death, neurological deficits	High
Ding 2015^[30]^	US	1989–2009	Cohort study	65.6 (60.3–71.0) mo	37.3	Male (n = 139)	270	Radiosurgery	46	102	94	28		Obliteration, intracranial hemorrhage, symptomatic RIC, permanent RIC	Medium
Ding 2015^[31]^	US	1989–2013	NRCT	78.7 ± 51.8 mo	12.6 ± 3.6	Male (n = 27)	51	Radiosurgery						Obliteration, radiologic RIC, symptomatic RIC, permanent RIC, post-radiosurgery cyst formation	Medium
Morgan 2015^[29]^	Finland	1989–2014	Cohort study	3.0y (6d–14.1 yr)	33.0 ± 13.0	Male (n = 64)	137	Surgery						Obliteration, neurologic deficits, stroke or death	High
Potts 2015^[28]^	US	1997–2013	NRCT	1.2 yr (2wk–12.8 yr)	39.4 ± 15.3	Male (n = 42)	112	Surgery	37	75				Obliteration, stroke or death, mRS score	Medium
Wang 2014^[32]^	Taiwan	1994–2005	NRCT	104 ± 98.5 mo	28 ± 28.07	Male (n = 66)	42	Radiosurgery						Obliteration, adverse effect, intracranial hemorrhage	Medium
Mohr 2014^[6]^	Germany	2007–2013	RCT	32.9 ± 19.2 mo	45 ± 12	Male (n = 66)	114	Endovascular treatment	32	44	28	8		Death or stroke, focal deficit, seizure, infection	High
Rutledge 2014^[8]^	US	2007–2013	RCT	21 mo	41	Male (n = 34)	15	Radiosurgery	8	21	21	7	3	Stroke or death, mRS score, obliteration	High
							43	Surgery							
							1	Endovascular treatment							
Ding 2013^[33]^	US	1989–2009	NRCT	86 ± 53 mo	36.9 ± 35.8	Male (n = 222)	444	Radiosurgery	81	167	163	30	3	Obliteration, intracranial hemorrhage, RIC, formation, radiation-induce, neoplasia, new-onset seizures	Medium
Yang 2012^[34]^	Korea	1997–2006	NRCT	89.9 mo			161	Radiosurgery						Obliteration, seizures	Medium

N = number, RIC = radiation-induced change.

### 3.2 Primary outcome

**Figure 2:**
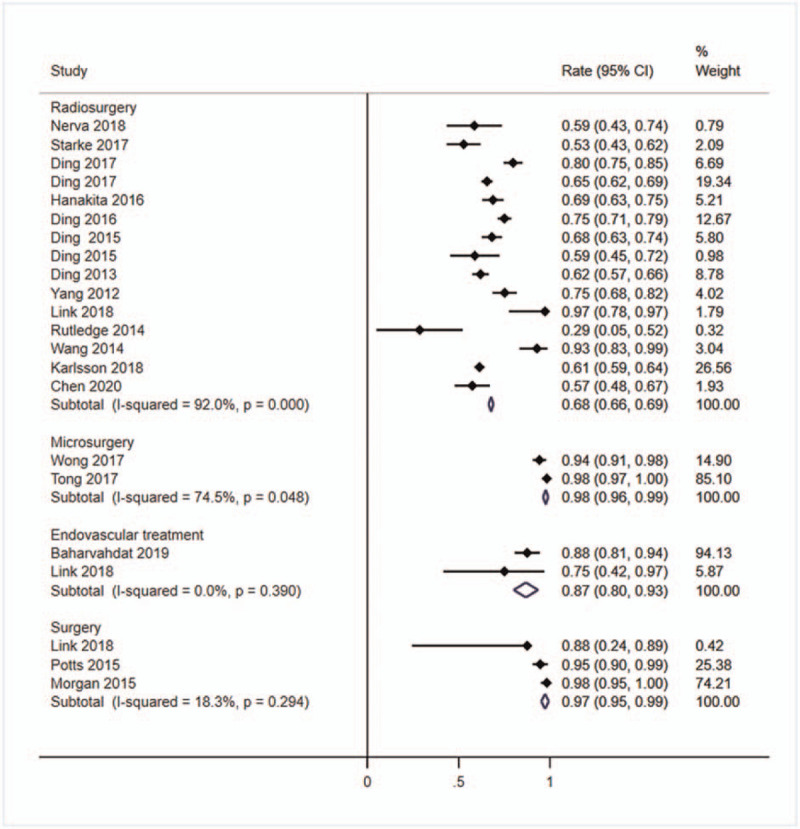
Forest plot of the pooled obliteration rate. Studies reported the obliteration rate of unruptured bAVMs patients after treating were pooled categorized by therapy methods. bAVMs = brain arteriovenous malformations.

Twenty-one studies^[1–3,7,11,16,19–21,23,24,27,28,30–37]^ with a total of 5452 patients with unruptured bAVMs reported the obliteration rate after treatment. The highest rate occurred in microsurgery group (*r* = 98%, 95%CI: 96%–99%, *I*^2^ = 74.5%), followed by surgery group (*r* = 97%, 95%CI: 95%–99%, *I*^2^ = 18.3%) and endovascular treatment group (*r* = 87%, 95%CI: 80%–93%, *I*^2^ = 0.0%), and lowest in radiosurgery group (*r* = 68%, 95%CI: 66%–69%, *I*^2^ = 92.0%) (Fig. 2).

### 3.3 Secondary outcomes

#### 3.3.1 Stroke/death

**Figure 3:**
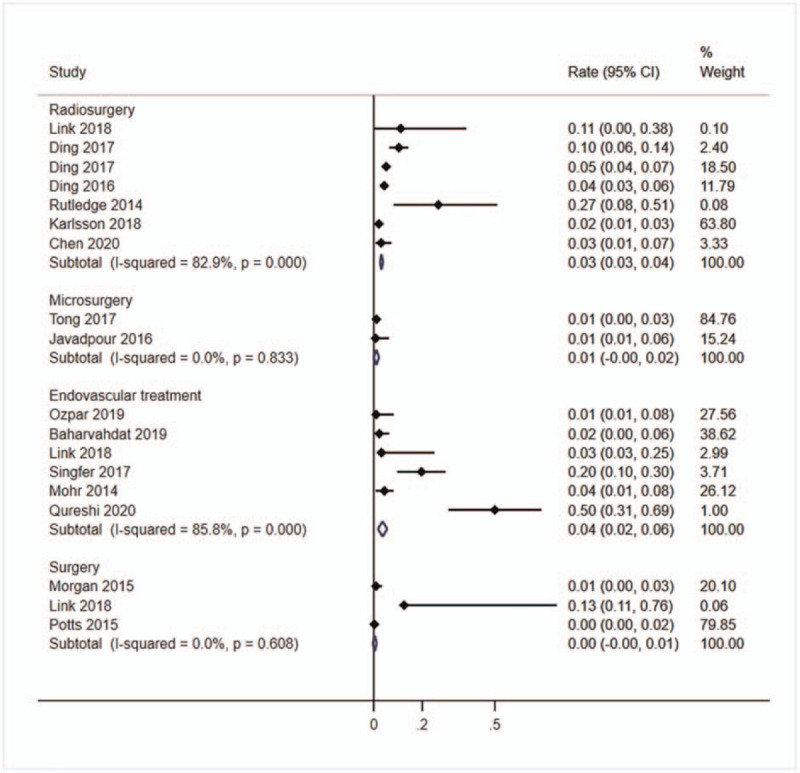
Forest plot of the pooled stroke/death rate. Studies reported the stroke/death rate of unruptured bAVMs patients after treating were pooled categorized by therapy methods. bAVMs = brain arteriovenous malformations.

Fifteen studies^[3,5,7,16–18,20,21,25,27–32]^ with a total of 3874 patients reported the occurrence of stroke or death after treatment. The highest rate of stroke or death was observed in the endovascular treatment group (*r* = 4%, 95%CI: 0%–8%, *I*^2^ = 85.8%), followed by the radiosurgery group (*r* = 3%, 95%CI: 3%–4%, *I*^2^ = 82.9%) and microsurgery group (*r* = 1%, 95%CI: 0%–2%, *I*^2^ = 0.0%), and lowest in the surgery group (*r* = 0%, 95%CI: 0%–1%, *I*^2^ = 0.0%) (Fig. 3).

#### 3.3.2 Hemorrhage

**Figure 4:**
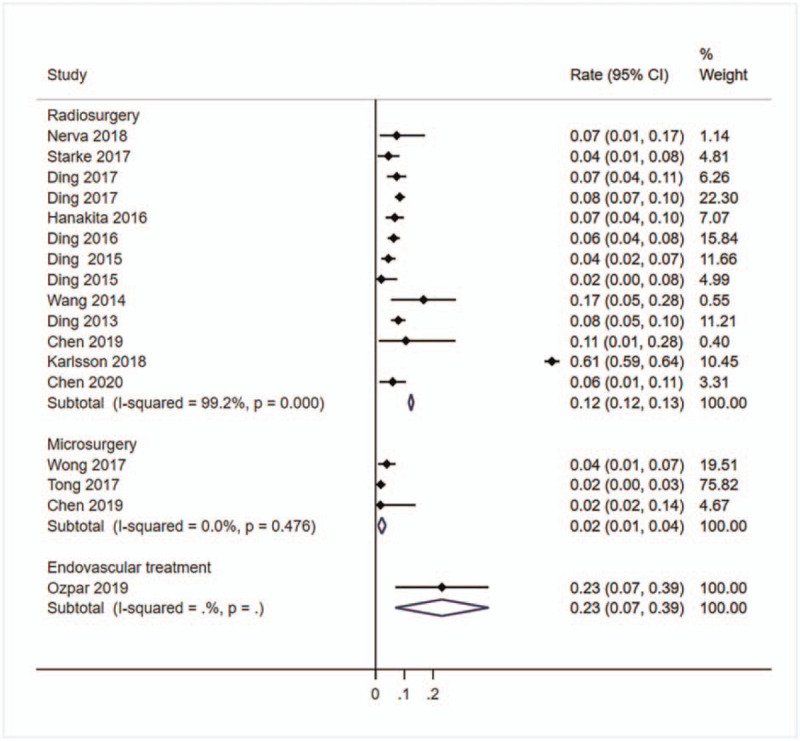
Forest plot of the pooled hemorrhage rate. Studies reported the hemorrhage rate of unruptured bAVMs patients after treating were pooled categorized by therapy methods. bAVMs = brain arteriovenous malformations.

Sixteen studies^[1,2,11,16,18,19,21–24,27,28,30,33,35,36]^ with a total of 4790 patients reported the hemorrhage rate after treatment. The highest rate was 23% in endovascular treatment group (95%CI: 7%–39%), followed by radiosurgery group (*r* = 12%, 95%CI: 12%–13%, *I*^2^ = 99.2%), and lowest in microsurgery group (*r* = 2%, 95%CI: 1%–4%, *I*^2^ = 0.0%) (Fig. 4).

#### 3.3.3 Neurological deficit

**Figure 5:**
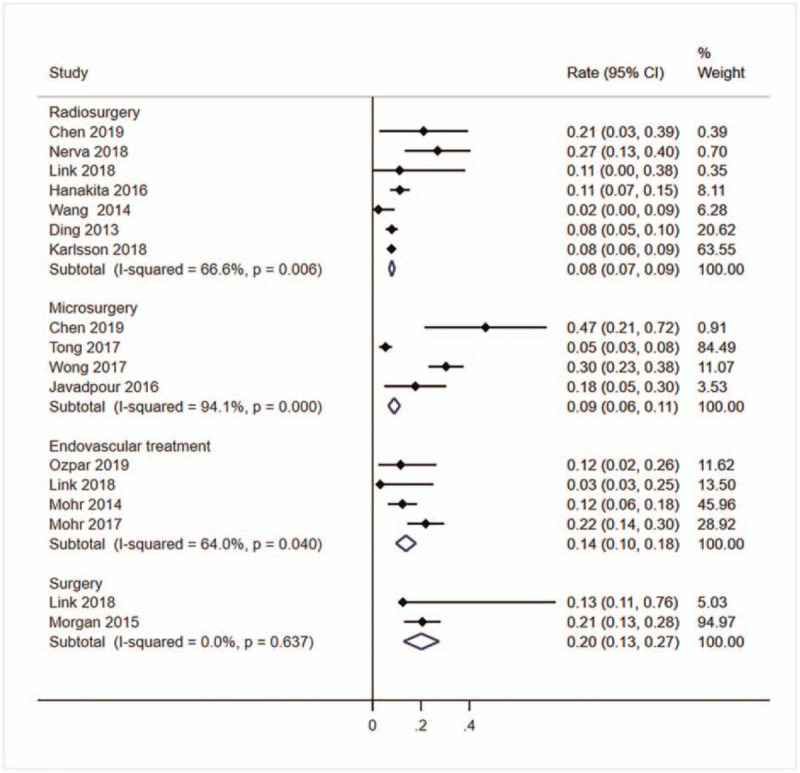
Forest plot of the pooled neurological deficit rate. Studies reported the neurological deficit rate of unruptured bAVMs patients after treating were pooled categorized by therapy methods. bAVMs = brain arteriovenous malformations.

Thirteen studies^[1–3,5,11,18,19,21,23,26,29,30,32]^ with a total of 3104 patients reported the occurrence of neurological deficit after treatment. The rate was highest in surgery group (*r* = 20%, 95%CI: 13%–27%, *I*^2^ = 0.0%), followed by endovascular treatment group (*r* = 14%, 95%CI: 10%–18%, *I*^2^ = 64.0%) and microsurgery group (*r* = 9%, 95%CI: 6%–11%, *I*^2^ = 94,1%), and lowest in radiosurgery group (*r* = 8%, 95%CI: 7%–9%, *I*^2^ = 66.6%) (Fig. 5).

### 3.4 Tertiary outcomes

Other outcomes with their pooled rates were shown in Table 2. A 44% and 2% of patients who received endovascular treatment and radiosurgery developed epilepsy, respectively. In addition, 2% of patients who received microsurgery or endovascular treatment underwent postoperative infection. As to mRS score, most of the patients in the microsurgery group (88%) compared with the surgery group (81%) and endovascular treatment group (54%) received low scores (0–1) while patients in the endovascular treatment group (22%) rather than microsurgery group (12%) and surgery group (12%) received high scores (≥2). What's more, in the radiosurgery group, 4% of patients had permanent RIC, 34% had radiologic RIC, and 11% had symptomatic RIC.

**Table 2:** Meta-analyses of pooled rates of other outcomes after treatment.

					Heterogeneity	
Outcomes	Treatment	Included studies (n)	Patients (n)	Pooled rate (95%CI)	(*I*^2^, %)	*P* value
Epilepsy	Radiosurgery	4	801	2% (0%–4%)	71.6	.014
	Endovascular treatment	1	114	44% (35%53%)	–	–
Postoperative infection	Microsurgery	1	282	2% (0%–4%)	–	–
	Endovascular treatment	1	114	2% (0%–5%)	–	–
mRS–<–2	Microsurgery	2	316	88% (85%–92%)	62.4	.103
	Endovascular treatment	2	87	54% (46%–62%)	98.0	<.001
	Surgery	1	112	81% (74%–88%)	–	–
mRS–≥–2	Microsurgery	2	316	12% (8%–15%)	61.2	.108
	Endovascular treatment	2	87	22% (13%–30%)	0.0	.693
	Surgery	1	112	12% (6%–18%)	–	–
Permanent RIC	Radiosurgery	5	1930	4% (3%–5%)	90.9	<.001
Radiologic RIC	Radiosurgery	4	495	34% (30%–38%)	90.3	<.001
Symptomatic RIC	Radiosurgery	6	1981	11% (9%–12%)	0.0	.452

CI = confidence interval, mRS = modified Rankin Scale, n = number, RIC = radiation-induced change.

### 3.5 Subgroup analyses

#### 3.5.1 Continent

Continent-subgroup analysis showed that on obliteration, Asian patients had a higher proportion after radiosurgery (*r* = 79%, 95%CI: 65%–92%) and microsurgery treatment (*r* = 98%, 95%CI: 97%–100%), while European patients had a higher proportion after endovascular treatment (*r* = 88%, 95%CI: 81%–94%) and surgery (*r* = 98%, 95%CI: 95%–100%). As to stroke/death, Asian patients had a lower proportion after radiosurgery treatment (*r* = 1%, 95%CI: 0%–3%) while European patients had a lower proportion after endovascular treatment (*r* = 3%, 95%CI: 1%–6%). In addition, Asian patients had the lowest proportion of getting hemorrhage after microsurgery treatment (*r* = 2%, 95%CI: 0%–3%). As for the neurological deficit, Asian patients also had the lowest proportion after microsurgery treatment (*r* = 5%, 95%CI: 3%–8%) and radiosurgery treatment (*r* = 8%, 95%CI: 6%–9%) (Table S1, Supplemental Digital Content).

#### 3.5.2 Study duration, time of follow-up, study quality, study design, and publication characteristics

We also conducted subgroup analyses stratified by study duration, time of follow-up, study quality, study design, and publication characteristics. We found that studies with more than 10 years of duration reported a higher proportion of obliteration on radiosurgery treatment and a lower proportion of other complications (Table S2, Supplemental Digital Content). The same results could also be found in studies with a longer time of follow-up in the follow-up subgroup (Table S3, Supplemental Digital Content). In addition, a lower proportion of neurological deficit was observed in the microsurgery group with a longer time of follow-up (Table S3, Supplemental Digital Content). As to study quality subgroup, higher proportions of obliteration and other complications, and lower proportions of hemorrhage were found in high-quality studies (Table S4, Supplemental Digital Content). Studies with NRCT design seemed to report higher proportions of obliteration but lower complications (Table S5, Supplemental Digital Content). After stratified by publication characteristics, studies published in journals with high quality reported higher proportions of obliteration and hemorrhage, and lower proportions of stroke/death (Table S6, Supplemental Digital Content).

### 3.6 Meta-regression

Results of meta-regression showed that there was a statistical difference regarding hemorrhage rate among different continents on radiosurgery treatment (*P* = .023). However, we did not see other statistically significant results due to insufficient studies (number of studies) on specific groups (Table S7, Supplemental Digital Content).

### 3.7 Sensitivity analysis

Sensitivity analyses were conducted on treatment groups with high heterogeneity. Therefore, the radiosurgery group, microsurgery group, and endovascular treatment group were performed with sensitivity analysis. After the deletion of any single study, we could not see any statistically significant heterogeneity and association changes (Figure S1, Supplemental Digital Content).

### 3.8 Publication bias

Publication bias was needed to be examined in pooled results including 7 or more studies. Therefore, funnel plots and Egger tests were conducted to identify publication bias on the main outcomes of the radiosurgery group. We did not identify any significant statistical bias on obliteration rate (*P* = .805), stroke or death (*P* = .078), hemorrhage (*P* = .948), and neurological deficit (*P* = .161) (Figure S2, Supplemental Digital Content).

## 4 Discussion

The bAVMs are congenital anomalies of blood vessels that cause intracranial hemorrhage. However, congenital factors cannot fully explain the growth of arteriovenous malformations. Studies have confirmed that the biological behavior of bAVMs is closely related to the changes of high hemodynamic factors.^[38]^ The hemodynamic state of long-term high flow and low resistance directly causes vascular endothelial function damage, leading to a large number of inflammatory cell infiltration around arteriovenous malformations vessels, which leads to inflammatory reaction and oxidative stress reaction. Oxidative stress can play an important role in cerebrovascular diseases, possibly through the damaging of vascular endothelial function.^[39,40]^

It has been found that there is not only continuous oxidative stress but also a decrease of antioxidants in bAVMs patients. A study reported that when antioxidants decreased, oxidative stress caused more serious damage to malformed blood vessels, resulting in rupture and bleeding of malformed blood vessels.^[41]^ In addition, vascular endothelial growth factor (vascular endothelial growth factor, VEGF) and basic fibroblast growth factor (basic fibroblast growth factor, BFGF) also played an important role in angiogenesis. As a vascular growth factor, VEGF is mainly involved in the formation, growth, and differentiation of blood vessels. Studies have shown that VEGF plays a certain role in the growth of malformed vascular mass after embolization.^[42]^ In the process of promoting the pathological angiogenesis of bAVMs, more VEGF promotes more neovascularization. And the instability of neovascularization is the direct cause of the vascular mass rupture of bAVMs. Therefore, oxidative stress may stimulate the formation and growth of malformed blood vessels through ERK-HIF-VEGF pathway, and the instability of neovascularization further leads to rupture and bleeding of malformed blood vessels.

Regarding the treatment of unruptured bAVMs patients, prior work has documented the effectiveness of the treatment group in terms of death or stroke prevention.^[1,3,7]^ However, there were some shortages among these studies, including short-term duration and lacking analyses of the effects of different treatments on patient prognostic indicators. In this study, we systematically estimated the proportion of obliteration and complications after various treatments and found that although radiosurgery, surgery, and endovascular treatment have played essential roles in the management of unruptured bAVMs, microsurgery has the highest obliteration rate and the lowest hemorrhage rate. The findings of the present study provide a more reliable basis for clinical practice.

Microsurgery, as an essential treatment of unruptured bAVMs, provides immediate benefits, eliminates the risk of further lifetime hemorrhage,^[10]^ and maybe performed safely in appropriately selected patients. In recent years, studies^[43,44]^ reported hemorrhage rates of 0.18% after microsurgery treatment, 1.7% after radiosurgery treatment, and 1.7% after endovascular treatment, which were consistent with the results of our study. However, this was at the expense of a significantly higher risk of new neurological deficits in the microsurgery group. In other words, in the microsurgery group, the most common reason for failure was a new neurological deficit.^[19]^ However, 1 recent study implied that patients with neurological deficits after microsurgery could recover neurological function over time.^[11,19]^ Endovascular treatment, as a less invasive treatment for vascular diseases, is now widely applied worldwide. It is questioned because people realize that it is not easy to achieve complete elimination. As reported, the introduction of Onyx (Medtronic, Irvine, CA, USA),^[45]^ the latest agent, offered more controllable intranidal dispersion and increased the cure rate of unruptured bAVMs. However, the endovascular treatment also remained associated with substantial complications despite technological advances. A meta-analysis of Onyx^[46]^ has reported that it might increase the rate of permanent neurological deficits and mortality. Unfortunately, we also found that the highest hemorrhage rate (23%) was observed in the endovascular treatment group. The potential mechanism is that embolization can promote angiogenesis and increase radiation resistance or make nidus more irregular and diffuse, making it impossible to eliminate it routinely.^[47,48]^ Meanwhile, the obliteration rate after endovascular treatment was not as impressive as we would have expected. It may cut off the nidus before resection or reduce the volume of the nidus before stereotactic radiosurgery (SRS).^[49]^ Therefore, endovascular treatment is typically used as preoperative neoadjuvant therapy for either resection or SRS. As for radiosurgery, it is a routine treatment for unruptured bAVMs patients, although with controversial effectiveness and disadvantages.

Ding et al found in their study comprised 938 patients with unruptured bAVMs that bAVMs obliteration was achieved similarly in 65%.^[28]^ In our meta-analysis, we found that complete obliteration rates of bAVMs following a single SRS treatment was 68% demonstrated by digital subtraction angiography (DSA). The mechanism of bAVMs obliteration in the radiosurgery treatment mainly is because it makes progressive intimal thickening, thrombosis of irradiated vessels, and eventual occlusion of the vascular lumen.^[50]^ Moreover, radiosurgery can reduce the frequency and intensity of seizures via some intrinsic effects on the unruptured bAVMs nidus and surrounding areas.^[51,52]^ However, we unfortunately found that after radiosurgery, 4% of patients had permanent RIC, 34% had radiologic RIC, and 11% had symptomatic RIC, which was consistent with Flickinger findings.^[53]^ This might be concerned with the overuse of radiation doses in pursuit of greater obliteration. Besides, we found that surgical treatment was very useful in eliminating the risk of hemorrhage and reducing the volume of the nidus. However, Morgan et al found^[54]^ that the abnormal vessels observed with filling slowly in the DSA immediately after surgery were not always effortless, especially in the absence of apparent arteriovenous shunting. Therefore, although statistically significant, it is not easy to quantify the magnitude of these effects. This is because of the small number of patients and the wide CIs.

The early termination and the short follow-up time were the main limitations of the ARUBA trial, which could cast doubt on the results.^[1,6,27,55]^ Therefore, we analyzed the effect of different follow-up time on treatment outcomes. We found that after an extended follow-up, the risk of stroke or death increased after endovascular treatment, which might be related to the neurological defects and hemorrhage caused by their postoperative.^[56]^ Although the meta-analysis showed that patients in the high-duration subgroup after microsurgery had higher rates of neurological deficits and lower rates of obliteration, we could not draw a certain conclusion. Mainly because with the larger time-span of a study, the heterogeneity of included patients could be increasing. Moreover, medical technology in different periods may have an impact on the prognosis. Due to the immature microsurgical techniques in the earlier period, the occurrence of neurological defects in such patients receiving microsurgery may be higher.^[1,11,29]^ It seems there are inequalities in bAVMs treatment and clinical outcomes between people from different ethnic backgrounds.^[1,11,19,26]^ Continent-subgroup analysis in this study has shown that compared with European patients, Asian patients had a higher obliteration rate and a lower incidence of neurological deficit after microsurgery. However, limited to existing studies, whether Asian bAVMs patients benefit more after microsurgery remains to be confirmed by further high-volume, multicentered, and extensive follow-up controlled trials.

Currently, all of these treatments about unruptured bAVMs may result in high risks of adverse prognosis as we have found. Moreover, with the advent of the era of individual genome sequencing, genotype researches and risk factors assessment of arteriovenous malformation have become more in-depth. Therefore, the biological therapeutic method may be an option with great promise for unruptured bAVMs. Increasing evidence showed that the genetic and environmental factors could jointly affect the occurrence of AVMs. Thomas et al^[57]^ found that DNA methylation in genes connected to vascular development had a vital role in the development of unruptured bAVMs. Chen et al^[58]^ showed that the risk of bAVMs might be related to methylation of the CDKN2A gene. In addition, Nakamura et al^[59]^ suggested that activated macrophages might increase the angiogenesis of PDCN possibly leading to hemorrhage events in unruptured bAVMs. Furthermore, studies have shown that^[60,61,62]^ the increase of VEGF may affect the proliferation and migration ability of vascular endothelial cells, further hyperplasia of malformed blood vessels, or rupture and hemorrhage of blood vessels. Fortunately, VEGFR1 has an anti-angiogenic effect, which can resist angiogenesis induced by VEGF in arteriovenous malformations. Bevacizumab, as an anti-VEGF monoclonal antibody, may inhibit the formation of arteriovenous malformation in the epidermis and brain, and affects the progress of blood malformation vessels. We have reasons to believe that with the further development of unruptured bAVMs genetics, drug design in the future can treat unruptured bAVMs according to its biological characteristics.

The essential limitation of our meta-analysis were that the pooled data available from retrospective, single-center studies with inherent selection and treatment biases. Although the heterogeneity was present in the summary estimates, we have carried out subgroup analyses and sensitivity analyses to explore the potential sources of heterogeneity. But we could not identify the statistical significance by meta-regression among most specific subgroups due to insufficient studies in each group. Detailed clinical outcomes in terms of hemorrhage, stroke or death, and obliteration were unavailable in these studies.^[7,22]^ Meanwhile, selection of unruptured bAVMs patients for certain treatment options is likely to have been affected by brain AVM characteristics and patient,^[63]^ which may hamper comparison of treatments. Due to the lack of detailed data, we were unable to compare the obliteration rates of various treatments according to the Spetzler–Martin grade and AVM localization. In addition, due to the insufficient eligible studies, we failed to further analyze the effect of different combination therapies combination regimens on patients’ prognosis. Furthermore, many patients were unable to obtain detailed clinical follow-up because the participating institutions were each tertiary referral centers with varying degrees of rigor in follow-up neurology and functional assessment. We also could not determine how many deaths were caused by unruptured bAVMs in patients.

## 5 Conclusions

Unruptured bAVMs are complex vascular diseases with serious potential neurological complications or death, but the best therapeutic option for unruptured bAVMs patients is still disputed. In this study, we found that microsurgery might provide lasting clinical benefits in some unruptured bAVMs patients for its high obliteration rates and low hemorrhage. However, with the limitations of studies we included, there were still considerable risks and incomplete efficacy in the treatments of unruptured bAVMs. Further prospective multicenter and long-term follow-up controlled trials are essential to address these limitations.

## Author contributions

**Data curation:** Yongle Zhan.

**Investigation:** Jianmin Piao.

**Methodology:** Yu Jiang.

**Project administration:** Renjie Liu.

**Resources:** Zhongxi Yang, Pengcheng Liu.

**Software:** Pengcheng Liu.

**Writing – original draft:** Renjie Liu, Yongle Zhan, Yun Wei.

**Writing – review & editing:** Jianmin Piao, Xuan Chen, Yu Jiang.

## Supplementary Material

Supplemental Digital Content

## Supplementary Material

Supplemental Digital Content

## Supplementary Material

Supplemental Digital Content

## Supplementary Material

Supplemental Digital Content

## Supplementary Material

Supplemental Digital Content

## Supplementary Material

Supplemental Digital Content

## Supplementary Material

Supplemental Digital Content

## Supplementary Material

Supplemental Digital Content

## Supplementary Material

Supplemental Digital Content
