# Supplementary material for: Treatments of unruptured brain arteriovenous malformations: A systematic review and meta-analysis
Source: Medicine (Baltimore). 2021 Jun 25;100(25):e26352. doi: 10.1097/MD.0000000000026352 (PMC8238300; doi:10.1097/MD.0000000000026352)
Supplement: Supplemental Digital Content [file medi-100-e26352-s001.docx]

**Supplementary Table 1 Continent-subgroup analysis of primary and secondary outcomes**

| **Treatment** | **North America** | | | | **Europe** | | | | **Asia** | | | |
| --- | --- | --- | --- | --- | --- | --- | --- | --- | --- | --- | --- | --- |
|  | **Included studies (n)** | **Patients (n)** | **Pooled rate (95%CI)** | **H**  **(*I*^2^, %)** | **Included studies (n)** | **Patients (n)** | **Pooled rate (95%CI)** | **H**  **(*I*^2^, %)** | **Included studies (n)** | **Patients (n)** | **Pooled rate**  **(95%CI)** | **H**  **(*I*^2^, %)** |
| **Obliteration** | | | | | | | | | | | | |
| Radiosurgery | 11 | 2721 | 69% (67%~70%) | 90.4* | - | - | - | - | 4 | 1794 | 79% (65%~92%) | 95.5* |
| Microsurgery | - | - | - | - | 1 | 155 | 94% (91%~98%) | - | 1 | 282 | 98% (97%~100%) | - |
| Endovascular treatment | 1 | 8 | 75% (42%~97%) | - | 1 | 88 | 88% (81%~94%) | - | - | - | - | - |
| Surgery | 2 | 114 | 95% (90%~99%) | 0.0 | 1 | 112 | 98% (95%~100%) | - | - | - | - | - |
| **Stroke/death** | | | | | | | | | | | | |
| Radiosurgery | 6 | 1803 | 5% (4%~6%) | 62.8* | - | - | - |  | 1 | 1351 | 2% (1%~3%) | - |
| Microsurgery | - | - | - | - | 1 | 32 | 1% (1%~6%) | - | 1 | 282 | 1% (0%~3%) | - |
| Endovascular treatment | 4 | 121 | 5% (1%~8%) | 91.2* | 2 | 202 | 3% (1%~6%) | 0.0 | - | - | - | - |
| Surgery | 2 | 114 | 0% (0%~1%) | 0.0 | 1 | 112 | 1% (0%~3%) | - | - | - | - | - |
| **Hemorrhage** | | | | | | | | | | | | |
| Radiosurgery# | 10 | 2717 | 7% (5%~7%) | 42.9* | - | - | - | - | 3 | 1633 | 39% (37%~41%) | 99.7* |
| Microsurgery | 1 | 15 | 2% (2%~14%) | - | 1 | 155 | 4% (1%~7%) | - | 1 | 282 | 2% (0%~3%) | - |
| Endovascular treatment | 1 | 26 | 23% (7%~39%) | - | - | - | - | - | - | - | - | - |
| **Neurological deficit** | | | | | | | | | | | | |
| Radiosurgery | 4 | 513 | 19% (6%~11%) | 66.9* | - | - | - | - | 3 | 1633 | 8% (6%~9%) | 75.9* |
| Microsurgery | 1 | 15 | 47% (21%~72%) | - | 2 | 189 | 27% (21%~34%) | 64.9* | 1 | 282 | 5% (3%~8%) | - |
| Endovascular treatment | 3 | 148 | 13% (1%~24%) | 74.7* | 1 | 114 | 12% (6%~18%) | - | - | - | - | - |
| Surgery | 1 | 2 | 10% (11%~75%) | - | 1 | 112 | 21% (13%~28%) | - | - | - | - | - |
| H: Heterogeneity, *: *p* < 0.10, #: *p* <0.05, difference was examined by meta-regression | | | | | | | | | | | | |
