# Supplementary material for: Treatments of unruptured brain arteriovenous malformations: A systematic review and meta-analysis
Source: Medicine (Baltimore). 2021 Jun 25;100(25):e26352. doi: 10.1097/MD.0000000000026352 (PMC8238300; doi:10.1097/MD.0000000000026352)
Supplement: Supplemental Digital Content [file medi-100-e26352-s002.docx]

**Supplementary Table 2 Duration-subgroup analysis of primary and secondary outcomes**

| **Treatment** | **≤10 years** | | | | **>10 years** | | | |
| --- | --- | --- | --- | --- | --- | --- | --- | --- |
|  | **Included studies (n)** | **Patients (n)** | **Pooled rate**  **(95%CI)** | **H**  **(*I*^2^, %)** | **Included studies (n)** | **Patients (n)** | **Pooled rate (95%CI)** | **H**  **(*I*^2^, %)** |
| **Obliteration** | | | | | | | | |
| Radiosurgery | 3 | 216 | 70% (64%~76%) | 87.7* | 8 | 1666 | 71% (69%~74%) | 92.2* |
| Microsurgery | 1 | 282 | 98% (97%~100%) | - | 1 | 155 | 94% (91%~98%) | - |
| Endovascular treatment | - | - | - | - | 2 | 96 | 87% (80%~93%) | 0.0 |
| Surgery | - | - | - | - | 3 | 226 | 97% (94%~100%) | 18.3 |
| **Stroke/death** | | | | | | | | |
| Radiosurgery | 1 | 15 | 27% (5%~48%) | - | 3 | 618 | 4% (3%~6%) | 0.0 |
| Microsurgery | 1 | 282 | 1% (0%~3%) | - | 1 | 34 | 1% (1%~6%) | - |
| Endovascular treatment | 2 | 140 | 3% (0%~5%) | 38.9 | 3 | 157 | 1% (0%~7%) | 81.3* |
| Surgery | - | - | - | - | 3 | 226 | 0% (0%~1%) | 0.0 |
| **Hemorrhage** | | | | | | | | |
| Radiosurgery | 1 | 41 | 7% (0%~15%) | - | 8 | 1676 | 6% (5%~7%) | 43.0* |
| Microsurgery | 1 | 282 | 2% (0%~3%) | - | 2 | 170 | 3% (1%~6%) | 0.0 |
| Endovascular treatment | 1 | 26 | 23% (7%~39%) | - | - | - | - | - |
| **Neurological deficit** | | | | | | | | |
| Radiosurgery | 1 | 41 | 27% (13%~40%) | - | 5 | 754 | 8% (6%~10%) | 61.7* |
| Microsurgery | 1 | 282 | 5% (3%~8%) | - | 3 | 204 | 28% (22%~35%) | 59.9* |
| Endovascular treatment | 3 | 254 | 16% (9%~22%) | 53.1 | 1 | 8 | 3% (3%~25%) | - |
| Surgery | - | - | - | - | 2 | 114 | 20% (13%~27%) | 0.0 |
| H: Heterogeneity, *: *p* < 0.10 | | | | | | | | |
