# Supplementary material for: Treatments of unruptured brain arteriovenous malformations: A systematic review and meta-analysis
Source: Medicine (Baltimore). 2021 Jun 25;100(25):e26352. doi: 10.1097/MD.0000000000026352 (PMC8238300; doi:10.1097/MD.0000000000026352)
Supplement: Supplemental Digital Content [file medi-100-e26352-s003.docx]

**Supplementary Table 3 Follow-up-subgroup analysis of primary and secondary outcomes**

| **Treatment** | **≤34 months** | | | | **>34 months** | | | |
| --- | --- | --- | --- | --- | --- | --- | --- | --- |
|  | **Included studies (n)** | **Patients (n)** | **Pooled rate**  **(95%CI)** | **H**  **(*I*^2^, %)** | **Included studies (n)** | **Patients (n)** | **Pooled rate (95%CI)** | **H**  **(*I*^2^, %)** |
| **Obliteration** | | | | | | | | |
| Radiosurgery | 3 | 961 | 68% (65%~70%) | 95.7* | 12 | 3554 | 68% (66%~69%) | 91.4* |
| Microsurgery | - | - | - | - | 2 | 437 | 97% (93%~100%) | 74.5* |
| Endovascular treatment | 2 | 96 | 87% (80%~93%) | 0.0 | - | - | - | - |
| Surgery | 1 | 2 | 88% (55%~100%) | - | 2 | 224 | 97% (93%~100%) | 52.4 |
| **Stroke/death** | | | | | | | | |
| Radiosurgery | 4 | 1471 | 5% (4%~6%) | 38.4 | 3 | 1683 | 2% (2%~3%) | 88.0* |
| Microsurgery | - | - | - | - | 2 | 316 | 1% (0%~2%) | 0.0 |
| Endovascular treatment | 4 | 236 | 4% (1%~6%) | 87.1* | 1 | 61 | 20% (10%~30%) | - |
| Surgery | 1 | 2 | 13% (11%~76%) | - | 2 | 224 | 0% (0%~1%) | 0.0 |
| **Hemorrhage** | | | | | | | | |
| Radiosurgery | 1 | 938 | 8% (7%~10%) | - | 12 | 3412 | 13% (13%~14%) | 99.3* |
| Microsurgery | 1 | 15 | 2% (0%~8%) | - | 2 | 437 | 2% (1%~4%) | 31.4 |
| **Neurological deficit** | | | | | | | | |
| Radiosurgery | 1 | 9 | 11% (0%~30%) | - | 6 | 2137 | 8% (7%~9%) | 72.0* |
| Microsurgery | 1 | 15 | 47% (21%~72%) | - | 3 | 471 | 9% (6%~11%) | 95.3* |
| Endovascular treatment | 3 | 236 | 13% (4%~22%) | 75.5* | - | - | - | - |
| Surgery | 1 | 2 | 10% (11%~75%) | - | 1 | 112 | 21% (13%~28%) | - |
| H: Heterogeneity, *: *p* < 0.10 | | | | | | | | |
