# Supplementary material for: Treatments of unruptured brain arteriovenous malformations: A systematic review and meta-analysis
Source: Medicine (Baltimore). 2021 Jun 25;100(25):e26352. doi: 10.1097/MD.0000000000026352 (PMC8238300; doi:10.1097/MD.0000000000026352)
Supplement: Supplemental Digital Content [file medi-100-e26352-s004.docx]

**Supplementary Table 4 Quality-subgroup analysis of primary and secondary outcomes**

| **Treatment** | **Medium** | | | | **High** | | | |
| --- | --- | --- | --- | --- | --- | --- | --- | --- |
|  | **Included studies (n)** | **Patients (n)** | **Pooled rate**  **(95%CI)** | **H**  **(*I*^2^, %)** | **Included studies (n)** | **Patients (n)** | **Pooled rate (95%CI)** | **H**  **(*I*^2^, %)** |
| **Obliteration** | | | | | | | | |
| Radiosurgery | 12 | 3850 | 67% (66%~68%) | 92.4* | 3 | 624 | 72% (68%~75%) | 91.7* |
| Microsurgery | 2 | 437 | 97% (93%~100%) | 74.5* | - | - | - | - |
| Endovascular treatment | 1 | 8 | 75% (42%~97%) | - | 1 | 88 | 88% (81%~94%) | - |
| Surgery | 2 | 114 | 95% (90%~99%) | 0.0 | 1 | 112 | 98% (95%~100%) | - |
| **Stroke/death** | | | | | | | | |
| Radiosurgery | 4 | 1351 | 3% (2%~4%) | 89.5* | 3 | 624 | 4% (3%~6%) | 57.8* |
| Microsurgery | 1 | 282 | 1% (0%~3%) | - | 1 | 34 | 1% (1%~6%) | - |
| Endovascular treatment | 3 | 95 | 3% (0%~6%) | 83.2* | 3 | 228 | 4% (1%~6%) | 91.4* |
| Surgery | 2 | 114 | 0% (0%~1%) | 0.0 | 1 | 112 | 1% (0%~3%) | - |
| **Hemorrhage** | | | | | | | | |
| Radiosurgery | 10 | 3721 | 14% (13%~15%) | 99.4* | 3 | 629 | 6% (4%~8%) | 0.0 |
| Microsurgery | 1 | 282 | 2% (0%~3%) | - | 2 | 170 | 3% (1%~6%) | 0.0 |
| Endovascular treatment | 1 | 26 | 23% (7%~39%) | - | - | - | - | - |
| **Neurological deficit** | | | | | | | | |
| Radiosurgery | 6 | 2127 | 8% (7%~9%) | 68.7* | 1 | 19 | 21% (3%~39%) | - |
| Microsurgery | 1 | 282 | 5% (3%~8%) | - | 3 | 204 | 28% (22%~35%) | 59.9* |
| Endovascular treatment | 2 | 34 | 7% (0%~15%) | 1.8 | 2 | 228 | 17% (7%~26%) | 73.7* |
| Surgery | 1 | 2 | 10% (11%~75%) | - | 1 | 112 | 21% (13%~28%) | - |
| H: Heterogeneity, *: *p* < 0.10 | | | | | | | | |
