# Supplementary material for: Treatments of unruptured brain arteriovenous malformations: A systematic review and meta-analysis
Source: Medicine (Baltimore). 2021 Jun 25;100(25):e26352. doi: 10.1097/MD.0000000000026352 (PMC8238300; doi:10.1097/MD.0000000000026352)
Supplement: Supplemental Digital Content [file medi-100-e26352-s005.docx]

**Supplementary Table 5 Study design-subgroup analysis of primary and secondary outcomes**

| **Treatment** | **RCT** | | | | **NRCT** | | | | **Cohort** | | | |
| --- | --- | --- | --- | --- | --- | --- | --- | --- | --- | --- | --- | --- |
|  | **Included studies (n)** | **Patients (n)** | **Pooled rate (95%CI)** | **H**  **(*I*^2^, %)** | **Included studies (n)** | **Patients (n)** | **Pooled rate (95%CI)** | **H**  **(*I*^2^, %)** | **Included studies (n)** | **Patients (n)** | **Pooled rate**  **(95%CI)** | **H**  **(*I*^2^, %)** |
| **Obliteration** | | | | | | | | | | | | |
| Radiosurgery | 1 | 14 | 29% (5%~52%) | - | 9 | 1729 | 74% (72%~76%) | 91.0* | 5 | 2772 | 63% (61%~65%) | 70.2* |
| Microsurgery | - | - | - | - | 2 | 437 | 97% (93%~100%) | 74.5* | - | - | - | - |
| Endovascular treatment | - | - | - | - | 2 | 96 | 87% (80%~93%) | 0.0 | - | - | - | - |
| Surgery | - | - | - | - | 2 | 114 | 98% (95%~100%) | 0.0 | 1 | 112 | 98% (95%~100%) | - |
| **Stroke/death** | | | | | | | | | | | | |
| Radiosurgery | 1 | 15 | 27% (5%~48%) | - | 3 | 750 | 5% (4%~7%) | 74.6* | 3 | 2389 | 3% (2%~3%) | 85.6* |
| Microsurgery | - | - | - | - | 1 | 282 | 1% (0%~3%) | - | 1 | 34 | 1% (1%~6%) | - |
| Endovascular treatment | 2 | 140 | 6% (2%~10%) | 95.2* | 4 | 157 | 3% (0%~5%) | 75.1* | - | - | - | - |
| Surgery | - | - | - | - | 2 | 114 | 0% (0%~1%) | 0.0 | 1 | 112 | 1% (0%~3%) | - |
| **Hemorrhage** | | | | | | | | | | | | |
| Radiosurgery | - | - | - | - | 7 | 1559 | 7% (5%~8%) | 41.3 | 6 | 2791 | 17% (16%~19%) | 99.6* |
| Microsurgery | - | - | - | - | 2 | 437 | 2% (1%~4%) | 31.4 | 1 | 15 | 2% (0%~8%) | - |
| Endovascular treatment | - | - | - | - | 1 | 26 | 23% (7%~39%) | - | - | - | - | - |
| **Neurological deficit** | | | | | | | | | | | | |
| Radiosurgery | - | - | - | - | 5 | 776 | 8% (6%~10%) | 74.9* | 2 | 1370 | 8% (6%~9%) | 50.1 |
| Microsurgery | - | - | - | - | 2 | 437 | 8% (6%~11%) | 97.5* | 2 | 49 | 24% (12%~35%) | 75.2* |
| Endovascular treatment | 2 | 228 | 17% (7%~26%) | 73.7* | 2 | 34 | 7% (0%~15%) | 1.8 | - | - | - | - |
| Surgery | - | - | - | - | 1 | 2 | 10% (11%~75%) | - | 1 | 112 | 21% (13%~28%) | - |
| RCT = randomized controlled trial, NRCT = non-randomized controlled trial, H: Heterogeneity, *: *p* < 0.10 | | | | | | | | | | | | |
