# Supplementary material for: Treatments of unruptured brain arteriovenous malformations: A systematic review and meta-analysis
Source: Medicine (Baltimore). 2021 Jun 25;100(25):e26352. doi: 10.1097/MD.0000000000026352 (PMC8238300; doi:10.1097/MD.0000000000026352)
Supplement: Supplemental Digital Content [file medi-100-e26352-s006.docx]

**Supplementary Table 6 Publication characteristics-subgroup analysis of primary and secondary outcomes**

| **Treatment** | **JCR-Quartile: Q1~Q2** | | | | **JCR-Quartile: Q3~Q4** | | | |
| --- | --- | --- | --- | --- | --- | --- | --- | --- |
|  | **Included studies (n)** | **Patients (n)** | **Pooled rate**  **(95%CI)** | **H**  **(*I*^2^, %)** | **Included studies (n)** | **Patients (n)** | **Pooled rate (95%CI)** | **H**  **(*I*^2^, %)** |
| **Obliteration** | | | | | | | | |
| Radiosurgery | 11 | 2771 | 67% (65%~68%) | 90.6* | 4 | 404 | 76% (72%~80%) | 94.0* |
| Microsurgery | 1 | 155 | 94% (91%~98%) | - | - | - | - | - |
| Endovascular treatment | 1 | 88 | 88% (81%~94%) | - | 1 | 8 | 75% (42%~97%) | - |
| Surgery | 2 | 224 | 97% (95%~99%) | 52.4 | 1 | 2 | 88% (55%~100%) | - |
| **Stroke/death** | | | | | | | | |
| Radiosurgery | 5 | 1688 | 3% (2%~4%) | 81.1* | 2 | 241 | 10% (7%~14%) | 0.0 |
| Microsurgery | - | - | - | - | 2 | 316 | 1% (0%~2%) | 0.0 |
| Endovascular treatment | 5 | 313 | 4% (2%~6%) | 88.6* | 1 | 8 | 3% (0%~14%) | - |
| Surgery | 2 | 114 | 0% (0%~1%) | 0.0 | 1 | 112 | 13% (11%~76%) | - |
| **Hemorrhage** | | | | | | | | |
| Radiosurgery | 9 | 3936 | 14% (13%~15%) | 99.5* | 3 | 395 | 5% (3%~7%) | 54.6 |
| Microsurgery | 1 | 155 | 4% (1%~7%) | - | 1 | 282 | 2% (0%~3%) | - |
| Endovascular treatment | 1 | 26 | 23% (7%~39%) | - | - | - | - | - |
| **Neurological deficit** | | | | | | | | |
| Radiosurgery | 5 | 2118 | 8% (7%~9%) | 74.8* | 1 | 9 | 11% (0%~30%) | - |
| Microsurgery | 1 | 15 | 30% (23%~38%) | - | 2 | 316 | 6% (3%~8%) | 70.7* |
| Endovascular treatment | 3 | 254 | 16% (9%~22%) | 53.1 | 1 | 8 | 3% (3%~25%) | - |
| Surgery | 1 | 112 | 21% (13%~28%) | - | 1 | 2 | 10% (11%~75%) | - |
| **Treatment** | **Category: General Medicine** | | | | **Category: Specific Field** | | | |
|  | **Included studies (n)** | **Patients (n)** | **Pooled rate**  **(95%CI)** | **H**  **(*I*^2^, %)** | **Included studies (n)** | **Patients (n)** | **Pooled rate (95%CI)** | **H**  **(*I*^2^, %)** |
| **Obliteration** | | | | | | | | |
| Radiosurgery | - | - | - | - | 15 | 4515 | 68% (66%~69%) | 92.0* |
| Microsurgery | - | - | - | - | 1 | 155 | 94% (91%~98%) | - |
| Endovascular treatment | - | - | - | - | 2 | 96 | 87% (80%~93%) | 0.0 |
| Surgery | - | - | - | - | 3 | 226 | 97% (94%~100%) | 18.3 |
| **Stroke/death** | | | | | | | | |
| Radiosurgery | - | - | - | - | 7 | 3154 | 3% (3%~4%) | 82.9* |
| Microsurgery | - | - | - | - | 2 | 316 | 1% (0%~2%) | 0.0 |
| Endovascular treatment | 2 | 140 | 3% (0%~5%) | 38.9 | 4 | 183 | 5% (2%~8%) | 90.8* |
| Surgery | - | - | - | - | 3 | 226 | 0% (0%~1%) | 0.0 |
| **Hemorrhage** | | | | | | | | |
| Radiosurgery | - | - | - | - | 12 | 4331 | 12% (12%~13%) | 99.3* |
| Microsurgery | - | - | - | - | 2 | 437 | 2% (1%~4%) | 31.4 |
| Endovascular treatment | 1 | 26 | 23% (7%~39%) | - | - | - | - | - |
| **Neurological deficit** | | | | | | | | |
| Radiosurgery | - | - | - | - | 6 | 2127 | 8% (7%~9%) | 68.7* |
| Microsurgery | - | - | - | - | 3 | 471 | 9% (6%~11%) | 95.3* |
| Endovascular treatment | 2 | 140 | 12% (7%~18%) | 0.0 | 2 | 122 | 16% (10%~22%) | 86.7* |
| Surgery | - | - | - | - | 2 | 114 | 20% (13%~27%) | 0.0 |
| JCR = Journal Citation Reports, Q = quartile, H: Heterogeneity, *: *p* < 0.10 | | | | | | | | |
