# Supplementary material for: Treatments of unruptured brain arteriovenous malformations: A systematic review and meta-analysis
Source: Medicine (Baltimore). 2021 Jun 25;100(25):e26352. doi: 10.1097/MD.0000000000026352 (PMC8238300; doi:10.1097/MD.0000000000026352)
Supplement: Supplemental Digital Content [file medi-100-e26352-s007.docx]

**Supplementary Table 7 Meta-regression results of subgroups**

| **Treatment** | **Obliteration** | | | **Stroke/death** | | | **Hemorrhage** | | | **Neurological deficit** | | |
| --- | --- | --- | --- | --- | --- | --- | --- | --- | --- | --- | --- | --- |
|  | **Studies (n)** | **β (SE)** | ***p*** | **Studies (n)** | **β (SE)** | ***p*** | **Studies (n)** | **β (SE)** | ***p*** | **Studies (n)** | **β (SE)** | ***p*** |
|  | **Continent** | | | | | | | | | | | |
| Radiosurgery | 15 | 0.04 (0.44) | 0.336 | 7 | -0.02 (0.02) | 0.310 | 13 | 0.11 (0.43) | 0.023 | 7 | -0.04 (0.03) | 0.246 |
| Microsurgery | - | - | - | - | - | - | 3 | -0.01 (0.01) | 0.588 | 4 | -0.21 (0.05) | 0.061 |
| Endovascular treatment | - | - | - | 6 | -0.13 (0.16) | 0.443 | - | - | - | 4 | -0.01 (0.11) | 0.964 |
| Surgery | 3 | 0.37 (0.24) | 0.373 | 3 | 0.01 (0.01) | 0.625 | - | - | - | - | - | - |
|  | **Duration** | | | | | | | | | | | |
| Radiosurgery | 11 | 0.15 (0.12) | 0.230 | 4 | -0.23 (0.11) | 0.176 | 9 | -0.01 (0.05) | 0.790 | 6 | -0.19 (0.82) | 0.085 |
| Microsurgery | - | - | - | - | - | - | 3 | 0.02 (0.02) | 0.486 | 4 | 0.23 (0.12) | 0.188 |
| Endovascular treatment | - | - | - | 5 | 0.05 (0.07) | 0.522 | - | - | - | 4 | -0.12 (0.08) | 0.259 |
| Surgery | - | - | - | - | - | - | - | - | - | - | - | - |
|  | **Follow-up** | | | | | | | | | | | |
| Radiosurgery | 15 | 0.00 (0.11) | 0.981 | 7 | -0.01 (0.04) | 0.761 | 13 | 0.03 (0.17) | 0.848 | 7 | -0.01 (0.13) | 0.928 |
| Microsurgery | - | - | - | - | - | - | 3 | 0.01 (0.03) | 0.866 | 4 | -0.29 (0.20) | 0.274 |
| Endovascular treatment | - | - | - | 5 | 0.06 (0.25) | 0.819 | - | - | - | - | - | - |
| Surgery | 3 | 0.93 (0.17) | 0.676 | 3 | -0.12 (0.17) | 0.598 | - | - | - | - | - | - |
|  | **Quality** | | | | | | | | | | | |
| Radiosurgery | 15 | -0.12 (0.10) | 0.242 | 7 | -0.09 (0.04) | 0.821 | 13 | -0.05 (0.11) | 0.640 | 7 | 0.12 (0.13) | 0.378 |
| Microsurgery | - | - | - | - | - | - | 3 | 0.02 (0.02) | 0.486 | 4 | 0.23 (0.12) | 0.188 |
| Endovascular treatment | - | - | - | 6 | 0.09 (0.16) | 0.617 | - | - | - | 4 | 0.10 (0.07) | 0.301 |
| Surgery | 3 | 0.04 (0.02) | 0.373 | 3 | 0.01 (0.01) | 0.625 | - | - | - | - | - | - |
|  | **Study design** | | | | | | | | | | | |
| Radiosurgery | 15 | -0.02 (0.08) | 0.769 | 7 | -0.05 (0.02) | 0.106 | 13 | 0.08 (0.09) | 0.357 | 7 | 0.01 (0.01) | 0.902 |
| Microsurgery | - | - | - | - | - | - | 3 | -0.01 (0.03) | 0.866 | 4 | 0.12 (0.19) | 0.581 |
| Endovascular treatment | - | - | - | 6 | -0.17 (0.15) | 0.316 | - | - | - | 4 | -0.10 (0.07) | 0.301 |
| Surgery | 3 | 0.04 (0.02) | 0.373 | 3 | 0.01 (0.01) | 0.625 | - | - | - | - | - | - |
|  | **Publication characteristics: JCR-Quartile** | | | | | | | | | | | |
| Radiosurgery | 15 | 0.06 (0.09) | 0.513 | 7 | 0.07 (0.03) | 0.070 | 12 | -0.09 (0.11) | 0.416 | 6 | 0.02 (0.14) | 0.886 |
| Microsurgery | - | - | - | - | - | - | - | - | - | 3 | -0.21 (0.10) | 0.292 |
| Endovascular treatment | - | - | - | 6 | -0.11 (0.22) | 0.649 | - | - | - | 4 | -0.12 (0.08) | 0.259 |
| Surgery | 3 | -0.09 (0.17) | 0.676 | 3 | 0.12 (0.17) | 0.598 | - | - | - | - | - | - |
|  | **Publication characteristics: Category** | | | | | | | | | | | |
| Radiosurgery | - | - | - | - | - | - | - | - | - | - | - | - |
| Microsurgery | - | - | - | - | - | - | - | - | - | - | - | - |
| Endovascular treatment | - | - | - | 6 | 0.14 (0.15) | 0.407 | - | - | - | 4 | 0.01 (0.09) | 0.889 |
| Surgery | - | - | - | - | - | - | - | - | - | - | - | - |

β: coefficient, SE: standard error
