# Supplementary material for: Treatments of unruptured brain arteriovenous malformations: A systematic review and meta-analysis
Source: Medicine (Baltimore). 2021 Jun 25;100(25):e26352. doi: 10.1097/MD.0000000000026352 (PMC8238300; doi:10.1097/MD.0000000000026352)
Supplement: Supplemental Digital Content [file medi-100-e26352-s008.pdf]

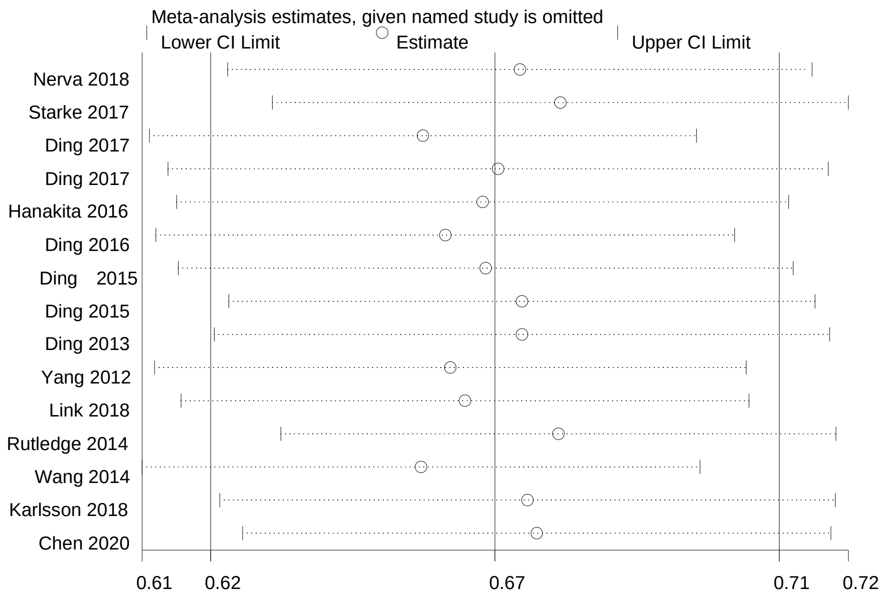

(a) Obliteration rate

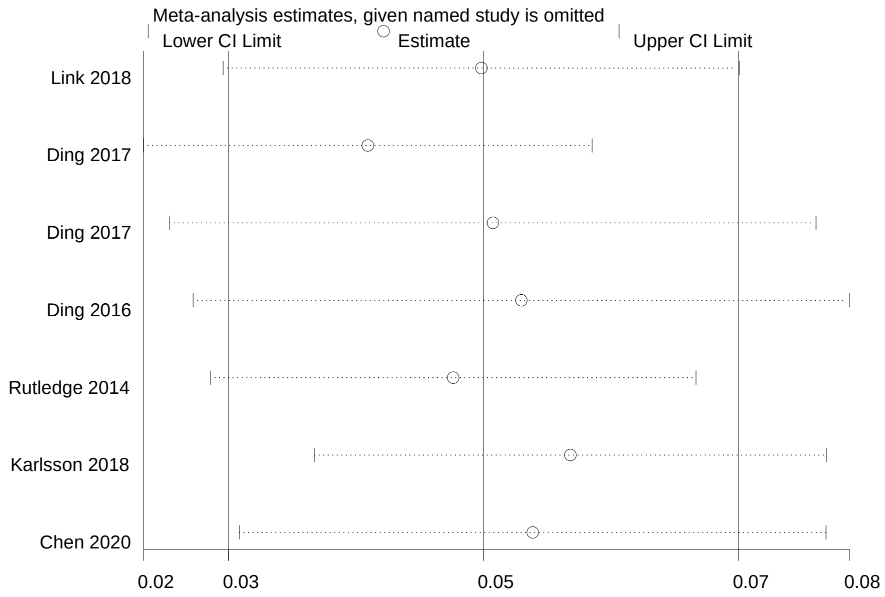

(b) Stroke or death

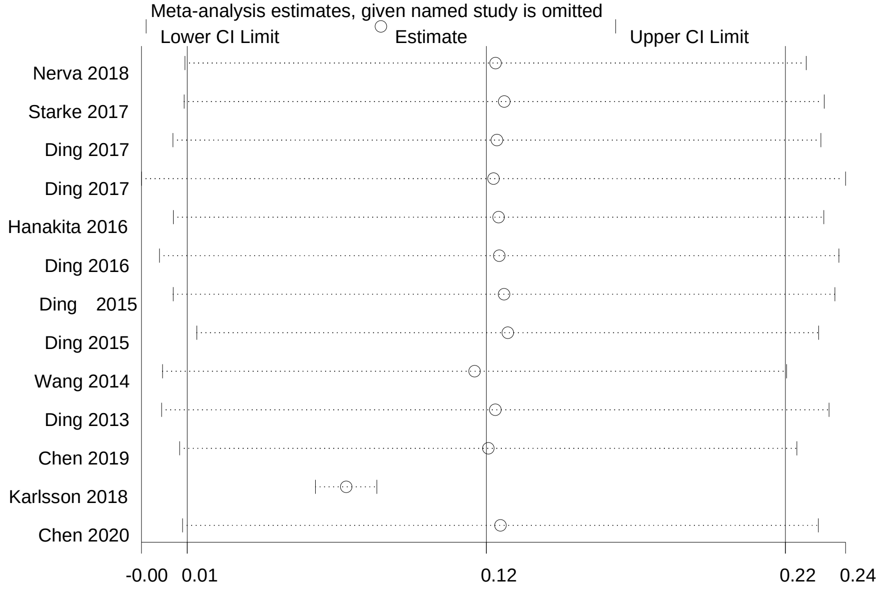

(c) Hemorrhage

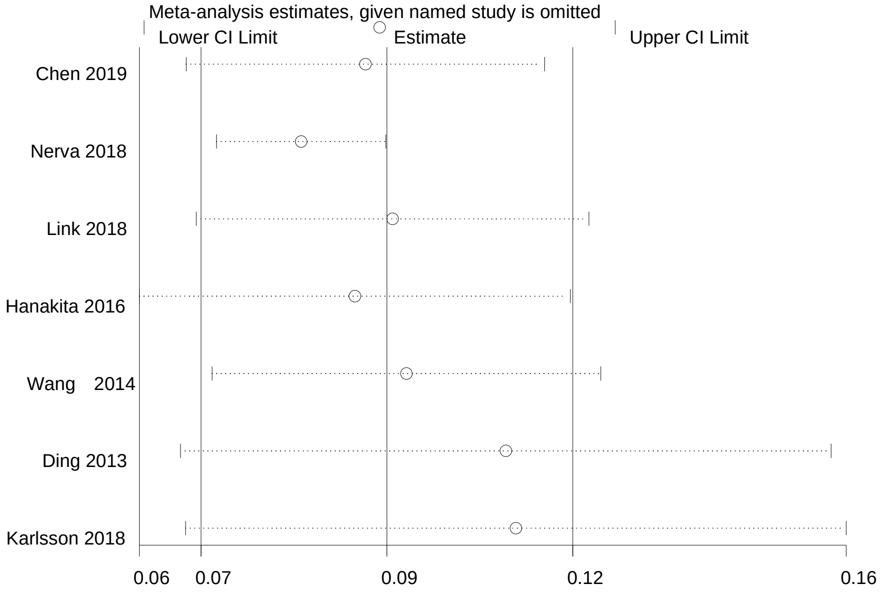

(d) Neurological deficit

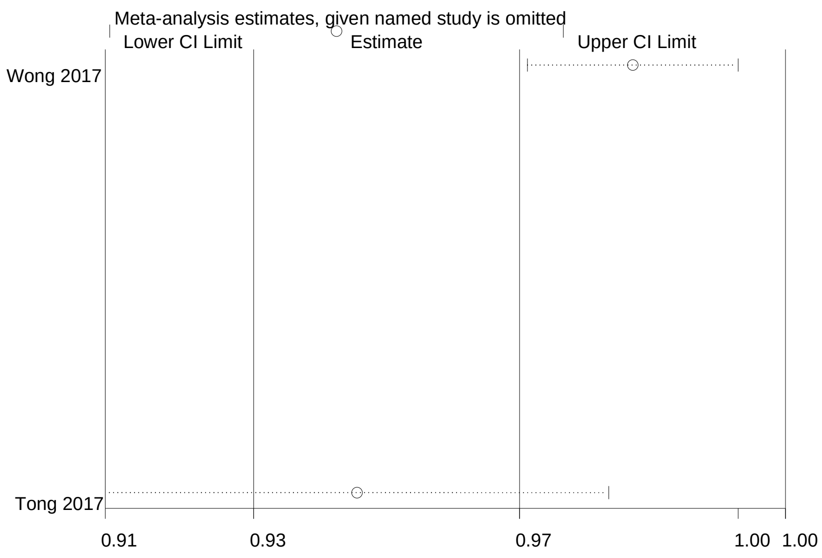

(e) Obliteration rate

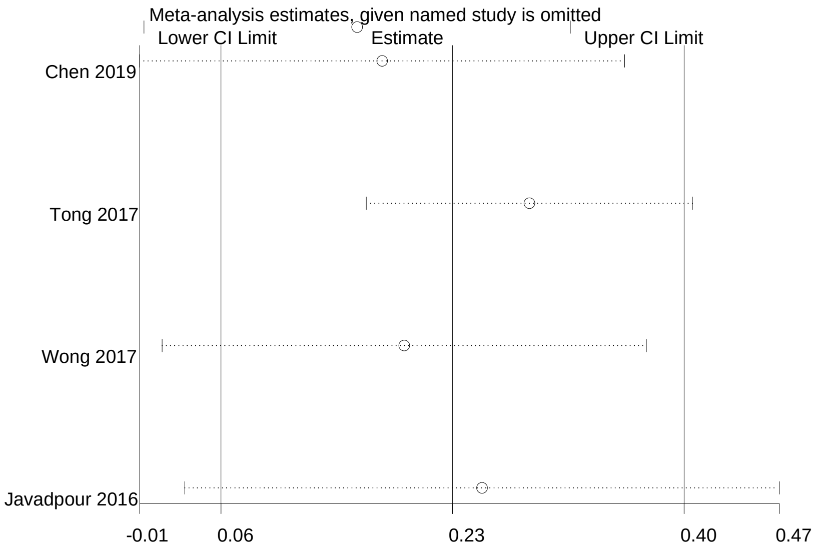

(f) Neurological deficit

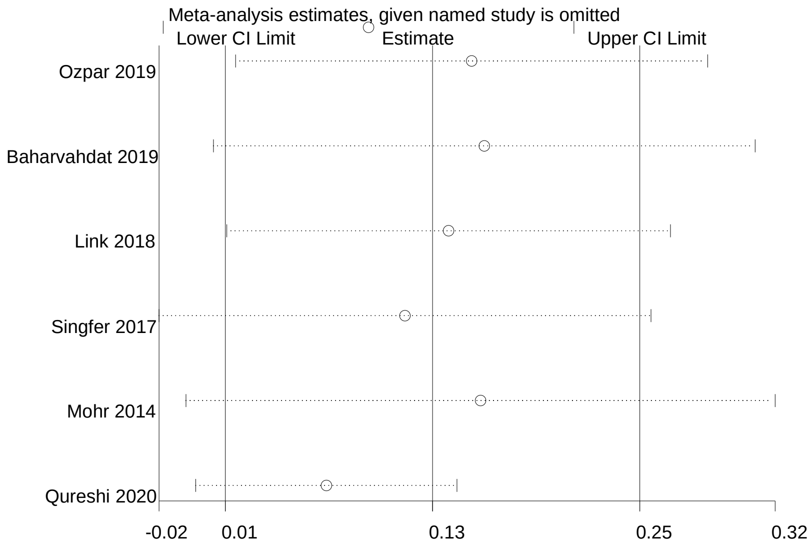

(g) Obliteration rate

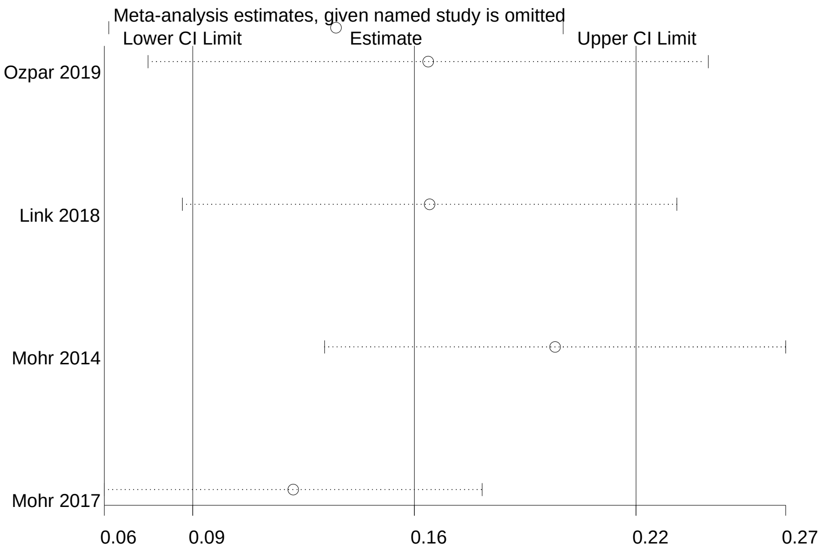

(h) Neurological deficit

Supplementary Figure 1. Sensitivity analyses

(a)~(d) radiosurgery group; (e)~(f) microsurgery group; (g)~(h) endovascular treatment group
