# Supplementary material for: Treatments of unruptured brain arteriovenous malformations: A systematic review and meta-analysis
Source: Medicine (Baltimore). 2021 Jun 25;100(25):e26352. doi: 10.1097/MD.0000000000026352 (PMC8238300; doi:10.1097/MD.0000000000026352)
Supplement: Supplemental Digital Content [file medi-100-e26352-s009.pdf]

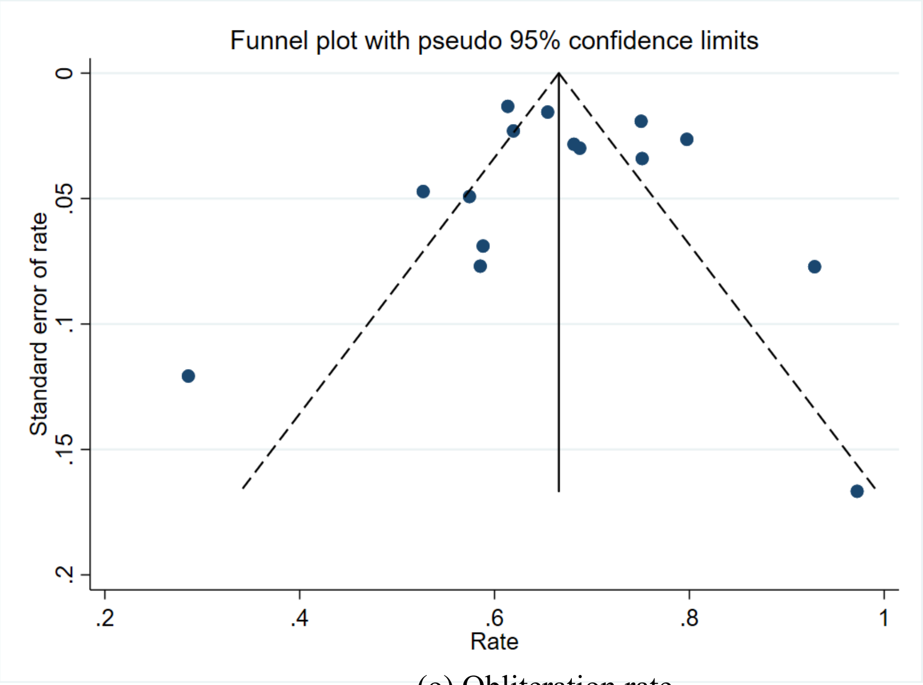

(o) Obliteration rate

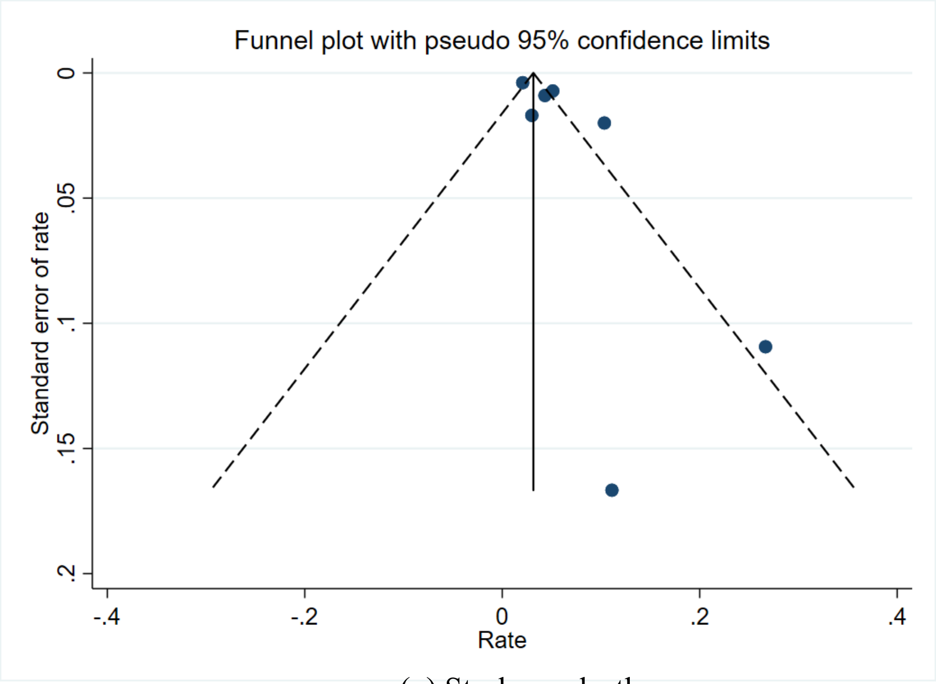

(p) Stroke or death

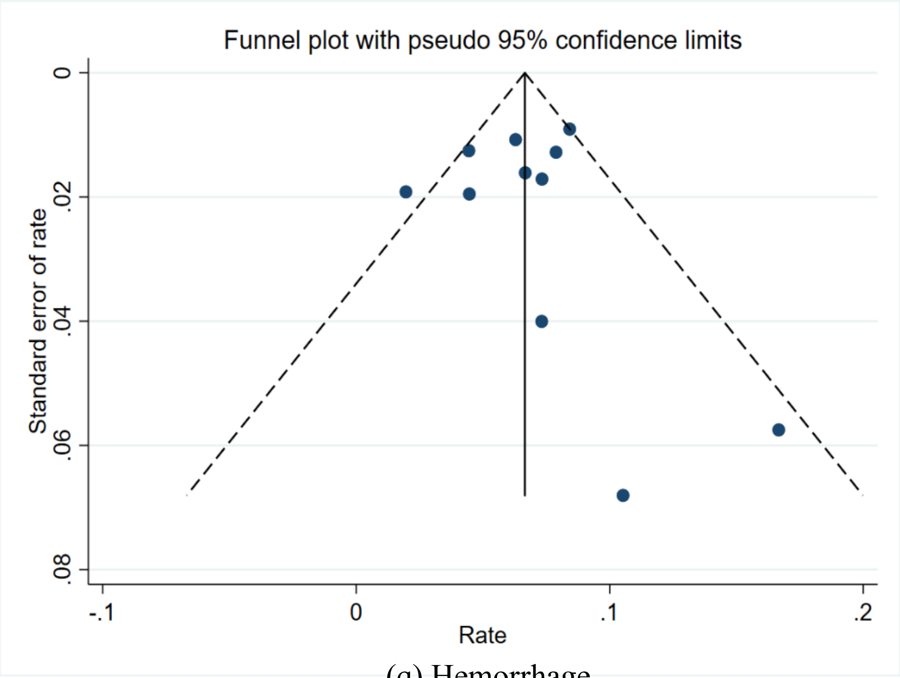

(q) Hemorrhage

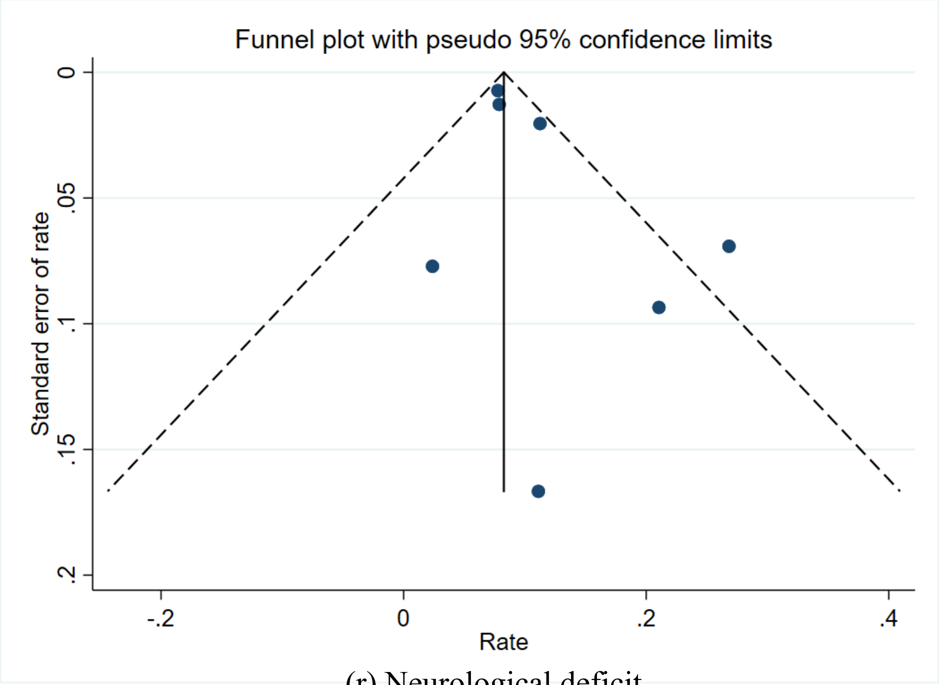

(r) Neurological deficit

Supplementary Figure 2. Funnel plots on radiosurgery group
